# How Chromatin Is Remodelled during DNA Repair of UV-Induced DNA Damage in *Saccharomyces cerevisiae*

**DOI:** 10.1371/journal.pgen.1002124

**Published:** 2011-06-16

**Authors:** Shirong Yu, Yumin Teng, Raymond Waters, Simon H. Reed

**Affiliations:** Department of Medical Genetics, Haematology, and Pathology, School of Medicine, Cardiff University, Cardiff, United Kingdom; The University of North Carolina at Chapel Hill, United States of America

## Abstract

Global genome nucleotide excision repair removes DNA damage from transcriptionally silent regions of the genome. Relatively little is known about the molecular events that initiate and regulate this process in the context of chromatin. We've shown that, in response to UV radiation–induced DNA damage, increased histone H3 acetylation at lysine 9 and 14 correlates with changes in chromatin structure, and these alterations are associated with efficient global genome nucleotide excision repair in yeast. These changes depend on the presence of the Rad16 protein. Remarkably, constitutive hyperacetylation of histone H3 can suppress the requirement for Rad7 and Rad16, two components of a global genome repair complex, during repair. This reveals the connection between histone H3 acetylation and DNA repair. Here, we investigate how chromatin structure is modified following UV irradiation to facilitate DNA repair in yeast. Using a combination of chromatin immunoprecipitation to measure histone acetylation levels, histone acetylase occupancy in chromatin, MNase digestion, or restriction enzyme endonuclease accessibility assays to analyse chromatin structure, and finally nucleotide excision repair assays to examine DNA repair, we demonstrate that global genome nucleotide excision repair drives UV-induced chromatin remodelling by controlling histone H3 acetylation levels in chromatin. The concerted action of the ATPase and C3HC4 RING domains of Rad16 combine to regulate the occupancy of the histone acetyl transferase Gcn5 on chromatin in response to UV damage. We conclude that the global genome repair complex in yeast regulates UV-induced histone H3 acetylation by controlling the accessibility of the histone acetyl transferase Gcn5 in chromatin. The resultant changes in histone H3 acetylation promote chromatin remodelling necessary for efficient repair of DNA damage. Recent evidence suggests that *GCN5* plays a role in NER in human cells. Our work provides important insight into how GG-NER operates in chromatin.

## Author Summary

Protection against genotoxic insult requires a network of DNA damage responses, including DNA repair. Inherited DNA repair defects cause severe clinical consequences including extreme cancer susceptibility. Despite detailed mechanistic understanding of the core reactions, little is known about the molecular events that initiate and regulate these processes as they occur in chromatin. We study the conserved nucleotide excision repair pathway in *Saccharomyces cerevisiae*. This pathway removes a broad spectrum of DNA damages including UV radiation–induced damage. Patients with mutations in genes involved in this process suffer dramatically elevated levels of skin and other cancers. Here we demonstrate how a group of genes involved in repair of transcriptionally silent regions of the genome, a process called global genome repair, modifies chromatin structure following UV irradiation to promote efficient removal of DNA damage from the genome. We show that the concerted action of global genome repair genes combine to regulate histone acetyl transferase accessibility to the chromatin in response to UV damage. In this way, global genome repair regulates histone H3 acetylation status, which ultimately regulates chromatin structure promoting efficient DNA repair in the genome. Our results contribute a significant advance in our understanding of how chromatin is processed during DNA repair.

## Introduction

DNA repair is a central facet of DNA metabolism, and nucleotide excision repair (NER) is an important component of a complex cellular response that prevents the loss of genetic information caused by DNA damage. Its importance for the repair of ultraviolet (UV) light induced DNA lesions is dramatically illustrated in humans who suffer from the autosomal recessive disease xeroderma pigmentosum (XP). Defective NER in these individuals severely predisposes them to sunlight-induced skin cancers [1]. The excision of lesions from non-transcribed regions of the human genome involves the global genome nucleotide excision repair (GG-NER) pathway, which in yeast requires the Rad7 and Rad16 GG-NER proteins [1]–[3]. Many of the core enzymatic activities associated with NER have been determined in some detail, but an understanding of how the process functions in relation to chromatin structure is still in its infancy.

DNA in eukaryotic cells is packaged into nucleosomes that form as a result of the wrapping of DNA around histone octamers. Higher-order chromatin structures are formed when nucleosomal arrays are further compacted. Chromatin has a major impact on DNA metabolic processes by controlling the functional interaction of proteins with regulatory and other elements in the DNA [4], [5]. Chromatin remodelling and histone modification are two major mechanisms that contribute to this regulation. Both processes have roles in controlling gene transcription [6], [7] and in NER [8]–[10].

GG-NER in *S.cerevisiae* requires both the Rad7 and Rad16 proteins [11]–[13]. Rad16 is a member of the SWI/SNF super-family of chromatin remodelling factors [14]. This superfamily of proteins exhibits ATPase activity that is stimulated by DNA or chromatin [15], [16], and all SWI/SNF-like proteins generate superhelical tension in linear DNA fragments *via* a DNA translocase activity associated with their ATPase function [17], [18]. The generation of superhelicity in DNA is a common mechanism of SWI/SNF-like chromatin remodelling complexes for altering chromatin structure [17]. We recently reported that a Rad7 and Rad16 containing protein complex also has DNA translocase activity. However, it is unable to slide nucleosomes unlike some SWI/SNF superfamily complexes [19]. Although Rad16 is a member of the SWI/SNF super-family, direct evidence of a role in chromatin remodelling is lacking. In this study we have addressed how GG-NER functions during DNA repair in chromatin in yeast cells.

UV irradiation stimulates histone H3 acetylation at lysine 9 and 14 (K9, K14) and chromatin remodelling, both globally and in the *MFA2* gene [8], [20]. However, these studies were not able to establish the precise relationship between these two events with respect to their effect on NER, nor did they inform on how these UV induced changes were regulated. Recently we showed that UV induced histone H3 acetylation depends on the Rad16 GG-NER protein. Furthermore, constitutively elevating histone H3 acetylation levels in the *MFA2* gene suppresses the requirement for Rad7 and Rad16 during GG-NER [10]. Gene regulation of *MFA2* involves the yeast general repressor complex Ssn6-Tup1 [21]. Deletion of *TUP1* results in constitutively elevated histone H3 acetylation and modified chromatin structure at the promoter of the *MFA2* gene [22]–[24]. Remarkably, Rad7 and Rad16 independent GG-NER occurs in the promoter region of *MFA2* in *TUP1* deleted cells. This suggested that Rad7 and Rad16 might regulate chromatin structure in response to UV damage during GG-NER via the regulation of histone H3 acetylation levels in chromatin.

In this report we demonstrate that the GG-NER proteins in yeast promote chromatin remodelling necessary for efficient DNA repair, revealing how this processes is regulated in response to DNA damage. We define a series of UV induced, Rad7 and Rad16 dependent events that control histone H3 acetylation which in turn drives chromatin remodelling necessary for efficient GG-NER in yeast. Histone H3 acetylation status at *MFA2* is determined by Rad7 and Rad16 controlling the occupancy of the Gcn5 histone acetyl transferase on chromatin in response to UV irradiation. These UV induced histone H3 modifications are required for chromatin remodelling necessary for efficient GG-NER in the region.

## Results

### UV-induced histone H3 acetylation (K9, K14) requires both Rad7 and Rad16

**Figure 1:**
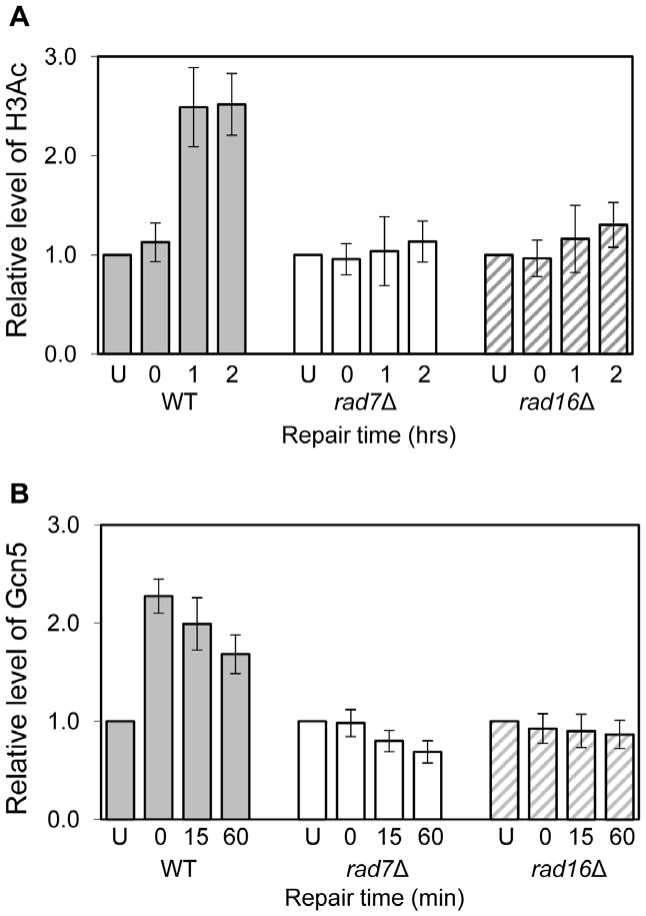
Histone H3 acetylation and occupancy of Gcn5 at the *MFA2* promoter. (A) ChIP analysis of Histone H3 acetylation (H3Ac) at the *MFA2* promoter using H3Ac (Lys 9 and Lys 14) antibody was performed in wild type (WT), *rad7Δ* and *rad16Δ* cells. U: untreated samples; 0: cells received 100 J/m^2^ of ultraviolet without repair; 1 and 2: cells were irradiated with ultraviolet and then allowed to repair in YPD medium for one or two hours respectively. Acetylation level shown is the fold change relative to unirradiated cells. Data are the average of at least three independent experiments ± SD. (B) ChIP with anti-myc antibody was performed in wild type, *rad7Δ* and *rad16Δ* cells. Gcn5 binding is presented as the fold change relative to untreated cells. Data are the average of at least three independent experiments ± SD.

Acetylation of histone H3 after UV irradiation depends on the presence of Rad16 and this process is necessary for efficient GG-NER [10]. Figure 1A shows that UV induced histone H3 acetylation (K9, K14) at the regulatory region of the *MFA2* gene also requires the GG-NER factor Rad7. Therefore Rad7 and Rad16 function in combination to increase histone H3 acetylation levels at *MFA2* in response to UV. Since UV induced histone H3 acetylation correlates with efficient GG-NER and elevated levels of histone H3 acetylation at *MFA2* suppress the requirement for Rad7 and Rad16 during GG-NER [10], this poses the question as to how Rad7 and Rad16 control histone H3 acetylation.

### Rad7 and Rad16 control histone H3 acetylation status by regulating Gcn5 occupancy at *MFA2*

Rad7 and Rad16 control UV induced histone H3 acetylation at *MFA2* and these proteins are not required for GG-NER when histone H3 acetylation is constitutively elevated in the region. We speculated that during GG-NER Rad7 and Rad16 mediate changes in histone H3 acetylation after UV by controlling the accessibility of the histone acetyl transferase Gcn5, which regulates histone H3 acetylation at *MFA2*. To test this we performed Gcn5 chromatin immunoprecipitation (ChIP) experiments in the promoter of the *MFA2* gene. Figure 1B shows the relative levels of Gcn5 binding at the repressed *MFA2* promoter in the absence of UV (U) or following UV irradiation at the times indicated (0, 15 and 60 minutes) in wild type, *rad7Δ* and *rad16Δ* strains. In the absence of UV irradiation, background levels of Gcn5 occupancy are detected in all three strains. However, after UV, a rapid increase in Gcn5 occupancy is observed in the wild type, but not in the *rad7Δ* or *rad16Δ* strains. In wild type cells, decreasing levels of Gcn5 occupancy at *MFA2* were observed with increasing time after UV irradiation and as repair occurred. Therefore in wild type cells Gcn5 occupies the promoter of the *MFA2* gene at low levels, resulting in background levels of histone H3 acetylation at *MFA2* in the absence of UV. Following UV, a Rad7 and Rad16 dependent increase in Gcn5 occupancy (Figure 1B) and histone H3 acetylation (Figure 1A) is observed at *MFA2*.

### Histone H3 acetylation regulates chromatin structure at the promoter of *MFA2*

**Figure 2:**
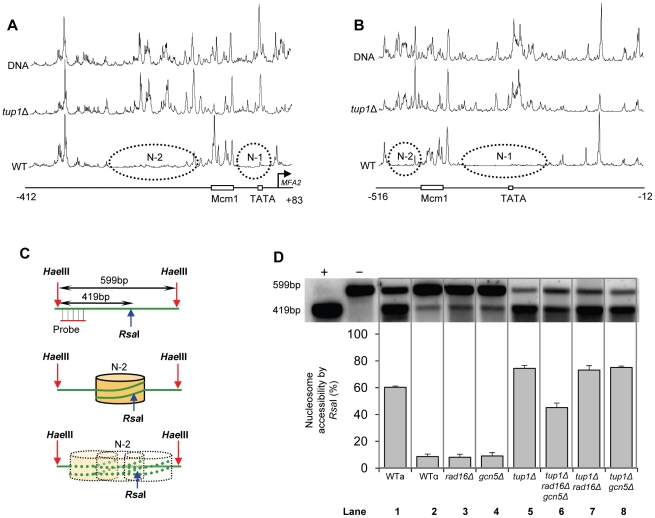
Densitometric scan of MNase sensitive regions of the *MFA2* promoter. (A and B) Relative MNase sensitivity is expressed graphically from scans of the gels shown in Figure S1. Trace A: transcribed strand (TS); Trace B: non-transcribed strand (NTS). The positioned nucleosomes observed in wild type cells are represented by ellipses N-1 and N-2. (C) Schematic representation of the assay in D. The middle of nucleosome N-2 of *MFA2* promoter has a single *Rsa*I restriction site within the *Hae*III restriction fragment. The probe shown detects either the full-length 599 bp of *Hae*III fragment or 419 bp of *Rsa*I and *Hae*III double digested fragment. The protection rendered by nucleosome N-2 limits the accessibility of *Rsa*I to the site. (D) Southern blot analysis of *Rsa*I accessibility to the *MFA2* promoter N-2 site. Lane −: naked DNA digested by *Hae*III only; lane +: naked DNA digested by both *Hae*III and *Rsa*I. Lanes 1–8 represent *Hae*III degisted DNA purified from *Rsa*I digested chromatin samples from the strains listed. The lower panel shows the data graphically.

We measured chromatin changes at *MFA2* in *TUP*1 deleted α-cells where histone H3 is hyperacetylated and where the requirement for Rad7 and Rad16 during GG-NER is abrogated. Tup1 is a component of a repressor complex that regulates gene expression at *MFA2*. In α mating type cells where the chromatin is repressed, the deletion of *TUP1* correlates with altered chromatin structure in *MFA2* and other *TUP1* regulated genes [10], [23], [25]. To confirm this we compared the MNase sensitive sites in naked DNA and chromatin from wild type and *tup1Δ* α-cells on both DNA strands of the *MFA2* promoter region (Figure 2A and 2B, Figure S1, and Text S1). Figure 2A and 2B reveal that MNase digestion is almost identical between *tup1Δ* α-cell chromatin and naked DNA, whereas chromatin from wild type α-cells exhibits significantly reduced MNase digestion due to protection by the positioned nucleosomes designated N-1 and N-2. Autoradiograms are shown in Figure S1. Therefore chromatin structure is altered in *TUP1* deleted α-cells. To further explore the effect of histone acetylation on chromatin structure we examined the accessibility of the restriction enzyme *Rsa*I to nucleosomal core DNA. Chromatin was treated with *Rsa*I restriction enzyme and purified DNA was digested using *Hae*III. Restriction with *Hae*III generated a 599 bp DNA fragment (Figure 2C). A double restriction digest with *Rsa*I and *Hae*III of naked DNA generated a smaller fragment of 419 bp (Figure 2C). In wild type α-cells *MFA2* is repressed by positioned nucleosomes and *Rsa*I has only limited access to the DNA at its restriction site located within nucleosome N-2. *Rsa*I digests only 8.7±1.9% of the total *MFA2* fragments (Figure 2D, Lane 2). However, in wild type a-cells and *tup1Δ* α-cells (Figure 2D, Lanes 1 and 5) where *MFA2* is derepressed, *Rsa*I cuts in both strains to the extent of 60.3±1.0% and 74.5±2.2% of the total *Hae*III fragments, respectively. Therefore, restriction enzyme sites are masked in chromatin from wild type α-cells, but are accessible in chromatin from wild type a-cells and *tup1Δ* α-cells.

### Increased histone H3 acetylation levels at *MFA2* in *TUP1* deleted α cells is dependent on Gcn5 and Rad16

**Figure 3:**
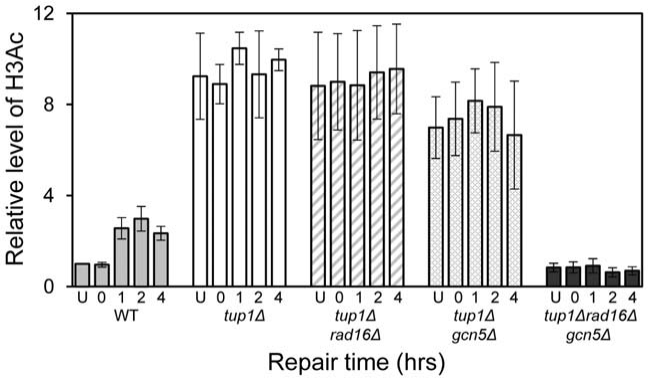
Histone H3 acetylation at the *MFA2*. ChIP analysis of Histone H3 acetylation (H3Ac) was performed using H3Ac (Lys 9 and Lys 14) antibodies. U: untreated samples; 0: cells received 100 J/m^2^ of ultraviolet without repair; 1, 2 or 4: cells were irradiated with ultraviolet and then were allowed to repair in YPD for the number of hours indicated. Acetylation level is presented as the fold change relative to unirradiated wild type cells. Data are the average of at least three independent experiments ± SD.

The relationship between chromatin accessibility and histone H3 acetylation status was examined by measuring the histone H3 acetylation levels in the *MFA2* promoter in the absence of and following UV irradiation. In Figure 1A, and in Figure 3, a three fold increased UV induced histone H3 acetylation is observed in wild type α-cells. In the *tup1Δα* strain an eight-fold elevation in constitutive histone H3 acetylation is observed and no further increase in H3 acetylation is seen following UV irradiation. A similar result was noted in *tup1Δrad16Δ α*-cells. Intriguingly, in *tup1Δgcn5Δ α*-cells histone H3 acetylation remains constitutively high, despite the loss of the Gcn5 histone acetyl transferase in this strain.

### Increased chromatin accessibility at *MFA2* in *TUP1* deleted α cells depends on Rad16 and Gcn5

Figure 2D lanes 3 and 4 demonstrate that in *RAD16* or *GCN5* deleted α-cells chromatin structure remains closed as evidenced by low-level *Rsa*I cutting observed (8.2%±2.3% and 9.0%±2.6% respectively), similar to levels seen in wild type α cells (Figure 2D, Lane 2). In *tup1Δrad16Δ* double mutant α-cells, open chromatin structure is retained as high levels of restriction enzyme cutting are observed (73.1%±3.4%) (Figure 2D, Lane 7), similar to levels seen in *tup1Δ α*-cells (Figure 2D, Lane 5). An open chromatin structure was also seen in *tup1Δgcn5Δ* α-cells shown in lane 8 (75.1%±1.0% *Rsa*I enzyme cutting). This was unexpected, since Gcn5 is deleted in this strain. But the result is consistent with the constitutively high histone H3 acetylation level detected (Figure 3), explaining the increased chromatin accessibility observed in this strain (Figure 2D, lane 8). Note that deleting *RAD16* in *tup1Δgcn5Δ* α-cells to create a *tup1Δrad16Δgcn5Δ*α triple mutant strain results in significantly reduced restriction enzyme cutting indicating the presence of a more repressive chromatin structure at the site (45.2%±3.4% *Rsa*I enzyme cutting) (Figure 2D, lane 6).

### Increased histone H3 acetylation levels and open chromatin structure are required for Rad7- and Rad16-independent GG-NER

**Figure 4:**
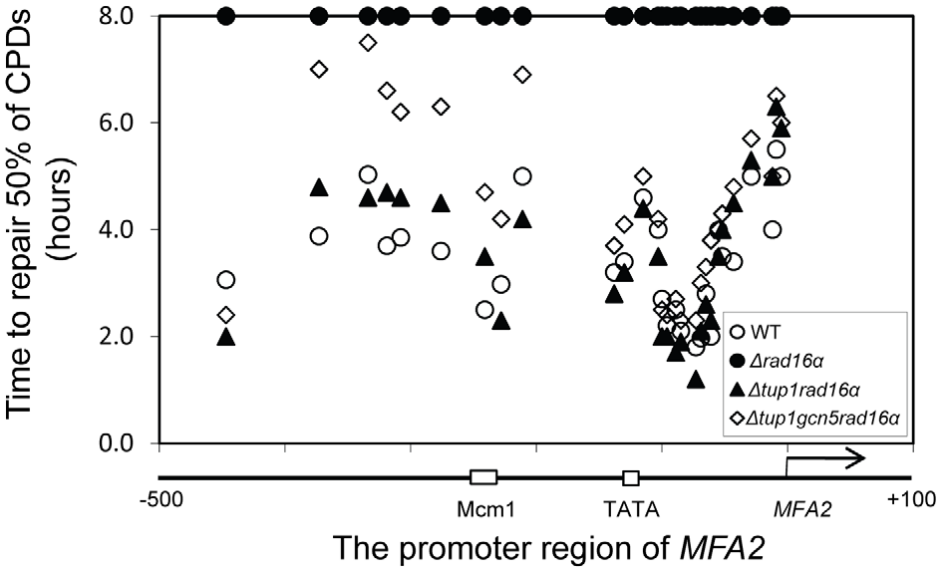
Repair of CPDs at the *MFA2* promoter. Time to remove 50% of the initial CPDs (T_50%_) at the sites indicated. T_50%_ of a single CPD or a clustered group of CPDs with similar repair rates was calculated as described previously (Teng *et al*, 2002) [20]. The T_50%_ of unrepaired CPDs (T_50%_≥8 h) were represented at the 8 h level on the graph. See also Figure S2.

Rad7 and Rad16 independent GG-NER occurs in genomic regions where constitutively elevated levels of histone H3 acetylation are observed, such as the promoter of *MFA2* in *tup1Δ* α-cells (Figure 4, Figure S2, and Text S1) [10]. The absence of CPD repair at *MFA2* in the *tup1Δ,rad14Δ* mutant proves that repair in the *tup1Δ,rad16Δ* α-cells occurs unequivocally via Rad7 and Rad16 independent GG-NER [10]. This suggested that Rad7 and Rad16 mediated UV induced histone H3 acetylation is necessary for efficient GG-NER. We examined this by measuring repair of CPDs in the promoter of *MFA2* in *tup1Δrad16Δ* α-cells and in *tup1Δrad16Δgcn5Δ* α-cells, where the histone acetyl transferase gene *GCN5* is deleted (Figure S2). Figure 4 shows the time taken to remove 50% of the CPDs (T_50%_) from the nontranscribed strand at the positions indicated. As seen previously, GG-NER in *tup1Δrad16Δ* α-cells, or *tup1Δrad7Δ*α is restored to near wild type levels compared to the lack of repair seen in the *rad16Δ* α single mutant cells (Figure 4). Therefore Rad7 and Rad16 are not required for GG-NER at *MFA2* when histone H3 acetylation levels are elevated creating an open chromatin structure (Figure 3 and Figure 2D, lane 5). To determine the significance of UV induced histone H3 acetylation levels and chromatin structure on efficient GG-NER we examined repair in *tup1Δrad16Δgcn5Δ* α-cells. Figure 4 reveals that loss of hisotne H3 acetylation which causes reduced chromatin accessibility [See Figure 3 and Figure 2D, lane 6] in this triple mutant strain, results in significantly reduced GG-NER in the region of nucleosomes N-1 and N-2 (see Figure 2A and 2B) upstream of the transcriptional start site (Figure 4: open diamonds). Repair in a small region in the vicinity of the transcriptional start site is unaffected. Therefore, the Rad7 and Rad16 independent GG-NER observed at *MFA2* in *TUP1* deleted cells is primarily due to the constitutively elevated levels of histone H3 acetylation and open chromatin structure in the region. We observed only background levels of histone H3 acetylation in the triple mutated strain and this results in a less accessible chromatin structure and reduced NER activity. This might imply that histone acetylation is not solely responsible for chromatin remodeling necessary for NER, because in the absence of detectable histone H3 acetylation, chromatin remains partially ‘open’. However our observations reveal that histone H3 acetylation does play a significant role in chromatin remodeling necessary for efficient NER. We also noted that in the absence of Gcn5, histone H3 acetylation at K9 and K14 can still be detected and this acetylation is dependent on Rad16, since acetylation is lost in the triple mutated strain (Figure 3). This underscores the significance of Rad16 in controlling histone acetylation status in the region, and demonstrates that redundancy exists with respect to the histone acetyl transferase that can be recruited to the chromatin. These observations are considered in more detail in the Discussion section.

### The ATPase and RING domains of Rad16 contribute to efficient UV survival

**Figure 5:**
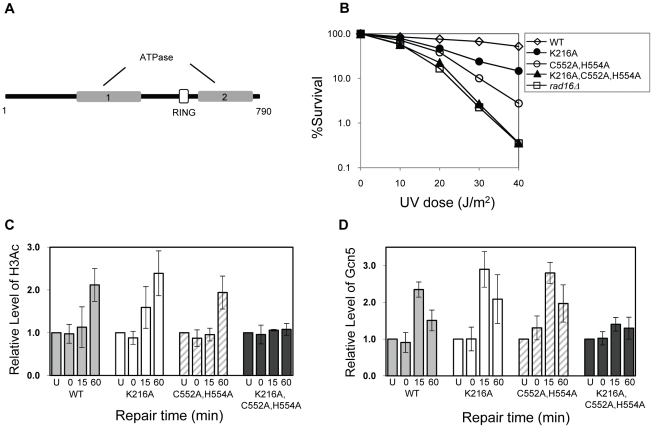
The effect of mutating specific domains in Rad16. (A) The domain structure of Rad16. (B) UV survival curves of the strains indicated. The result shows the average of three independent experiments. (C) Histone H3 acetylation at the *MFA2* promoter. ChIP analysis of Histone H3 acetylation (H3Ac) was performed using H3Ac (Lys 9 and Lys 14) antibody. U: untreated samples; 0: cells received 100 J/m^2^ of ultraviolet without repair; 15 and 60: cells were irradiated with UV and then were allowed to repair in medium for the times indicated. Acetylation level shown as the fold-change relative to unirradiated cells. Data are the average of at least three independent experiments ± SD. (D). The occupancy of Gcn5 at the *MFA2* promoter ChIP was performed with anti-myc antibody. Gcn5 binding is presented as the fold change relative to untreated cells. Data are represented as average of at least three independent experiments ± SD.

Rad16 has two known catalytic functions: a DNA translocase activity associated with the ATPase domain [19], and an E3 ubiquitin ligase activity associated with the C3HC4 RING domain embedded within the ATPase domain [see Figure 5A] [26], [27]. We introduced point mutations into each of the catalytic domains of Rad16 to examine their effect on GG-NER. The ATPase activity was tested by mutating the conserved Walker A box catalytic residue lysine 216 to alanine (K216A). This mutation creates an ATPase null mutant [27]. We call this the *RAD16* ATPase mutant. We also mutated the RING domain of Rad16 to test the role of the E3 ligase activity. RING domains have conserved cysteine and histidine residues that coordinate two zinc atoms. A conserved hydrophobic residue is also essential for the interaction between the RING domain and specific E2 ubiquitin conjugating enzymes. We made two point mutations in conserved cysteine and histidine residues; cysteine 552 to alanine and histidine 554 to alanine (C552A,H554A). We call this the *RAD16* RING mutant. Finally, we tested the effect of mutating both the ATPase and RING domains of Rad16 by introducing these mutations (K216A,C552A,H554A) into a single strain. Figure 5B compares the UV sensitivity in each of these strains compared to the parental wild type, and Rad16 deleted strains. The individual *RAD16* ATPase and RING mutant strains show intermediate UV sensitivity. Whereas the double mutant strain is as sensitive as the Rad16 deleted strain. These observations confirm previous findings that both the ATPase and RING E3 ligase catalytic activities contribute independently to efficient GG-NER and UV survival [27].

### The ATPase and RING domains of Rad16 are required for UV-induced Gcn5 occupancy and histone H3 acetylation

We examined the effect of the *RAD16* point mutations on the level of histone H3 acetylation and Gcn5 occupancy at *MFA2*. We performed histone H3 acetylation (K9, K14) ChIP experiments in the promoter of *MFA2*. Figure 5C shows the relative levels of acetylated histone H3 at the repressed *MFA2* in the absence of UV (U) or after UV irradiation at the times indicated (0, 15 and 60 minutes). In the absence of UV irradiation, background levels of histone H3 acetylation are detected in all four strains. However, following UV, a rapid increase in histone H3 acetylation is observed in the wild type strain and in the single *RAD16* ATPase and RING mutated strains, but not in the *RAD16* ATPase, RING double mutant strain, where UV induced histone H3 acetylation is abolished. Similar results were obtained when Gcn5 occupancy was examined in these strains, Figure 5D.

### The ATPase and RING domains of Rad16 are required for efficient GG-NER

**Figure 6:**
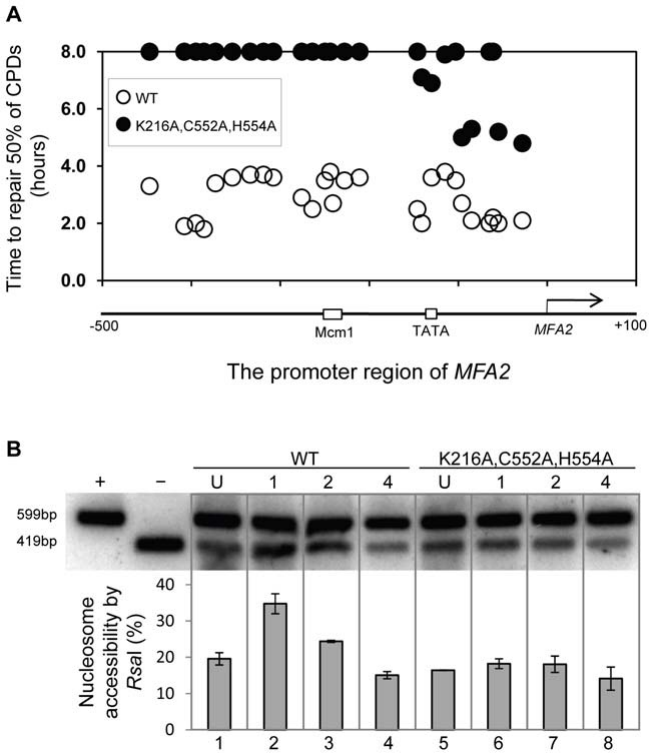
Repair of CPDs at the *MFA2* promoter. (A) Time to remove 50% of the initial CPDs (T_50%_) at given sites. T_50%_ of a single CPD or a clustered group of CPDs with a similar repair rate was calculated as described previously (Teng *et al*, 2002) [20]. The T_50%_ of unrepaired CPDs (T_50%_≥8 h) were represented at the 8 hour level on the graph. See also Figure S3. (B) Southern blot analysis of *Rsa*I accessibility to the *MFA2* promoter N-2 nucleosomal DNA, as described in legend to Figure 2D.

Finally, we examined the repair of CPDs at *MFA2* in wild type and each of the point mutated strains described above (Figure 6A, Figure S4, and Text S1). A typical autoradiogram is shown in Figure S3. In Figure 6A repair was expressed as the time taken to remove 50% of the CPDs (T_50%_) from the nontranscribed strand at the nucleotide positions indicated. As seen previously, GG-NER in the nontranscribed strand of *MFA2* proceeds efficiently in wild type cells (Figure 6A). Mutating either the ATPase domain or the RING domain of *RAD16* individually impairs UV lesion removal, but GG-NER continues less efficiently (Figure S4 and Text S1). This correlates with the near wild type levels of histone H3 acetylation, Gcn5 occupancy (Figure 5C and 5D), and intermediate UV sensitivity (Figure 5B) of these strains. GG-NER in the ATPase, RING domain double mutated strain is abolished over almost the whole of the *MFA2* promoter region and occurs at a level seen in the *RAD16* deleted strain (Figure 6A and Figure 4). This correlates with the lack of UV induced histone H3 acetylation and Gcn5 occupancy in the region (Figure 5C and 5D), and the high level of UV sensitivity (Figure 5B) observed in this strain. These observations demonstrate that the ATPase and RING domains of Rad16 function in combination to regulate UV induced Gcn5 occupancy and histone H3 acetylation status, which ultimately controls chromatin structure at *MFA2* in response to DNA damage. In Figure 6B we demonstrate the lack of UV induced chromatin remodelling observed in the ATPase, RING double mutated strain compared to the remodelling observed in the wild type strain using the restriction enzyme accessibility assay described earlier in Figure 2C and 2D. This confirms the importance of chromatin remodelling to the GG-NER process.

## Discussion

We've shown that Rad7 and Rad16 proteins are required for UV induced histone H3 acetylation at *MFA2*. These GG-NER factors regulate the acetylation status by controlling the occupancy of the histone acetyl transferase Gcn5 at this locus. In unirradiated wild type cells only background levels of Gcn5 are detected at *MFA2*, whereas increased Gcn5 occupancy is seen following UV irradiation. This correlates with increased acetylation of histone H3 observed in wild type cells in response to UV. In Rad7 and Rad16 deleted cells no increased Gcn5 occupancy or increased histone H3 acetylation is observed at *MFA2* in response to UV. This indicates that both events are Rad7 and Rad16 dependent in wild type cells. Increased histone acetylation levels have long been associated with changes in chromatin structure, particularly with respect to generating an open chromatin structure needed for gene transcription [8]. To address the impact of histone H3 acetylation on chromatin structure at *MFA2* in response to UV, we employed two methods: a nucleosome mapping assay, and a restriction enzyme accessibility assay. We examined these events in *TUP1* deleted cells since Tup1 is a component of the Ssn6-Tup1 general repressor complex, which regulates gene expression in a range of genes including *MFA2*. In α mating type yeast cells *MFA2* is repressed, but in *TUP1* deleted α-cells histone H3 levels at *MFA2* are constitutively elevated which results in an open chromatin structure at *MFA2*. We found that cells with elevated levels of histone H3 acetylation as is the case when *TUP1* alone, *TUP1,RAD16* and *TUP1,GCN5* are deleted in α-cells also have an open chromatin structure as demonstrated in the restriction enzyme accessibility assay in Figure 2D. We were surprised to detect elevated levels of histone H3 acetylation, and open chromatin structure in *TUP1,GCN5* deleted cells since the histone acetyl transferase Gcn5 known to function at *MFA2* in wild-type cells is absent in this strain [8]. We speculate that in *GCN5* deleted cells, an alternative histone acetyl transferase can substitute for *GCN5*. Significantly, this redundancy is dependent on Rad16, since in *tup1Δrad16Δgcn5Δ* triple mutant cells, histone H3 acetylation is reduced to background levels and the open chromatin structure is altered to a more repressed state. We suggest that these observations underscore the significance of Rad16 in regulating the histone acetylation status of chromatin in the region, and indicate that Rad16 determines histone acetyl transferase recruitment to the chromatin. We examined the significance of histone H3 acetylation at *MFA2* on lesion removal during GG-NER by measuring repair in *TUP1* deleted cells. To determine whether the elevated levels of histone H3 acetylation and open chromatin structure observed in *TUP1,RAD16* deleted cells promotes the repair observed in these cells, we examined repair in the *tup1Δrad16Δgcn5Δ* triple mutant strain where histone H3 levels are diminished to background levels, and chromatin accessibility is significantly reduced. We found that the near wild type level of repair observed in the *TUP1,RAD16* deleted cells was significantly reduced in the *tup1Δrad16Δgcn5Δ* mutant cells indicating the importance of histone H3 acetylation and chromatin structure to the repair observed in the region. Despite detecting only background levels of histone H3 acetylation in the triple mutant strain, we still detect a partially open chromatin structure, which results in a reduced but not totally defective NER efficiency. Our findings demonstrate that UV induced histone H3 acetylation is playing an important role in chromatin remodelling during NER, but recognise that other factors might also be influencing the process.

**Figure 7:**
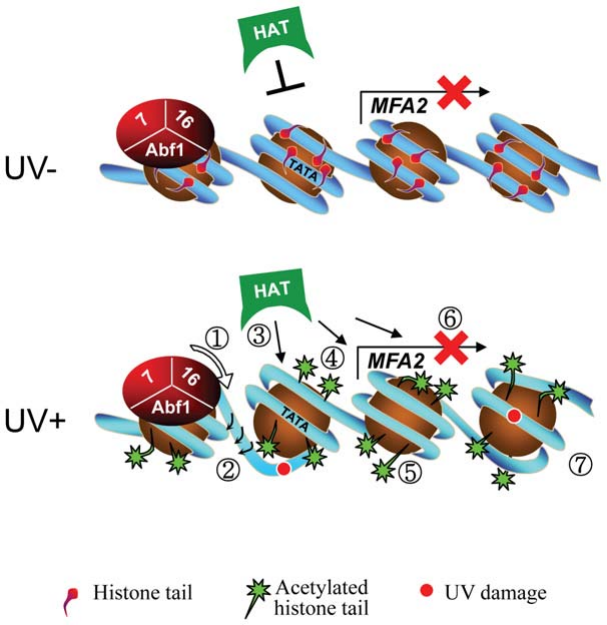
Model for UV-induced chromatin remodeling during GG-NER. Top panel. In the absence of UV, basal levels of histone acetyl transferase occupancy are detected on the chromatin of the MFA2 promoter. The absence of histone acetyl transferase occupancy is marked by the presence of an inhibitory link. Consequently, histone H3 tails remain unacetylated and chromatin remains repressed. Lower Panel. Following UV the DNA translocase (1) and E3 ligase (2) activities of Rad16 in the GG-NER complex promote increased histone acetyl transferase occupancy on chromatin as indicated by the presence of arrows (3) and histone H3 acetylation (4) that drives chromatin remodeling as shown by a more open chromatin structure around the nucleosomes (5). Failure of the GG-NER complex to slide nucleosomes may prevent transcription factor binding explaining the continued repression of *MFA2* transcription (6) despite chromatin remodeling. GG-NER dependent chromatin remodeling promotes efficient lesion removal (7).

Finally, we investigated whether either of the known activities associated with Rad16 was responsible for controlling this series of events. Strains carrying point mutations in the ATPase or the C3HC4 RING domain of Rad16, or a double mutant carrying both these mutations were examined. Previous studies showed that the Rad16 ATPase mutant has no detectable ATPase function [27], and the Rad16 RING mutant has no E3 ligase activity. UV survival experiments showed an intermediate UV sensitivity for the Rad16 ATPase and RING domain single mutants, while the double domain mutant showed higher UV sensitivity, similar to that observed in the Rad16 deleted strain (Figure 5). Therefore both the ATPase and E3 ligase functions of Rad16 are required for efficient GG-NER, in agreement with previous studies [26], [27]. This observation suggests that a UV induced ubiquitination event, possibly involving a histone or alternatively another NER factor, is likely important in initiating the chromatin remodelling process. It is established that UV induced histone ubiquitination is observed in human cells and is necessary for efficient NER [28]. We showed that both Rad16 domains contribute to efficient GG-NER. Figure S4 shows reduced levels of CPD removal from the nontranscribed strand of the *MFA2* promoter in each of the single domain mutant strains, and defective lesion removal only in the double domain mutant strain (Figure 6A). This observation correlates with the level of Gcn5 occupancy and histone H3 acetylation levels observed in these strains (Figure 5C and 5D). Loss of UV induced Gcn5 occupancy and histone H3 acetylation is only observed in the double mutant strain suggesting that the ATPase and RING domains of Rad16 are both required for efficient chromatin remodelling during GG-NER. Figure 6B confirms that efficient GG-NER observed in the wild type strain is dependent on UV induced chromatin remodelling since failure to remodel chromatin in the ATPase, RING double mutant strain results in defective repair. Collectively our results demonstrate that during GG-NER the Rad7 and Rad16 proteins promote efficient repair by regulating histone acetyl transferase occupancy on chromatin in response to UV. This explains how histone H3 acetylation status and chromatin structure is controlled in response to DNA damage, and that this process is necessary for efficient GG-NER. Our results are consistent with a model for UV induced chromatin remodelling in yeast cells described in Figure 7 (See Text S1 for further discussion).

It was recently reported that Gcn5 is recruited to sites of UV induced DNA damage in human cells [29]. However, its role in chromatin remodelling was not determined. Our studies provide important insight into how chromatin is remodelled to facilitate efficient DNA repair following UV induced DNA damage in human cells.

## Materials and Methods

### Plasmids and yeast strains

The details of plasmids and yeast strains used in this study can be found in Text S1 and in Table S1.

### UV survival assays

Cells were grown in synthetic complete medium with leucine dropout (SC-leu^−^) to mid-log phase (around 2×10^7^ cells/ml). Following mild sonication, cells were plated on SC-leu^−^ agar plates, then irradiated with the germicidal UV lamp at the indicated UV doses. Following irradiation, plates were immediately wrapped in foil and incubated for 3 days at 30°C. Survival was derived from the number of colonies relative to that in the unirradiated control. Experiments were performed in triplicate.

### Chromatin immunoprecipitation (ChIP)

This was performed as in Yu *et al*, [8] with modifications. In brief, proteins were cross-linked to DNA by addition of formaldehyde to 100 ml yeast cells (about 2×10^9^ cells) to a final concentration of 1% for 20 min at room temperature. 5.5 ml of Glycine (2.5 M) was added to stop cross-linking. Cells were lysed by the addition of 0.5 ml of glass beads (Sigma), and vortexed for 30 min on a Disruptor Genie at 4°C. The cell lysate was sonicated to generate DNA fragments ranging from 200–500 bps in length. Sonication was carried out using the Bioruptor (Diagenode) following the manufacturer's instruction at 4°C, power position “H”, 20 seconds on and 40 seconds off for 6 cycles. 50 µl of pre-washed pan mouse or anti-rabbit IgG Dynabeads was incubated with 2.5 µg of mouse anti-Myc (9E11, Abcam) antibody, or 2.5 µl of rabbit anti-acetyl histone H3 (at K9 and K14, Upstate Biotechnology) at 30°C for 30 min, then the antibody bound Dynabeads were subsequently incubated with 100 µl sheared chromatin solution equivalent to 10^8^ cells in a total volume of 0.5 ml for 3 hours at 21°C. After elution with pronase buffer (125 mM Tris pH 7.5, 25 mM EDTA, 2.5% SDS) from Dynabeads beads, formaldehyde cross-linking was reversed by incubating the eluate at 65°C overnight in the presence of 125 µg of pronase. Finally, DNA was purified with PCR purification kit (QIAGEN). 50 µl of chromatin solution was taken as input control for each sample. Quantitative PCR was performed in real time using iQ SYBR Green Supermix (Bio-Rad) and diluted DNA in the Bio-Rad MyiQ. PCR was performed in triplicate for each sample, and melting curves were executed to ensure single PCR products. Primers for amplifying nucleosome N-2 in the promoter region of *MFA2* are:

primer 1, AAAGCAGCATGTTTTCATTTGAAACA;
primer 2, TATGGGCGTCCTATGCATGCAC.

### Chromatin preparation, MNase digestion, and the high-resolution nucleosome mapping

These were carried out as described previously [30]

### Restriction enzyme accessibility

Chromatin was prepared as described in Teng *et al*, [30] with modifications. In brief, cells from 200 ml YPD (2–4×10^9^ cells) were pelleted, washed in cold PBS and 1 M Sorbitol, and spheroplasted in 1 m lysis solution (1 M Sorbitol, 5 mM 2-mercatoethanol) containing 20 mg of Zymolyase-20T per 1 g of cells for 20 min at 30°C. Spheroplasts were washed with cold 1 M Sorbitol, and lysed in 7 ml Ficoll solution (18% Ficoll, 20 mM KH_2_PO_4_, pH 6.8, 1 mM MgCl_2_, 0.25 mM EGTA, 0.25 mM EDTA) per 1 g cell. Collecting nuclei by centrifugation, and washing the pellet with *Rsa*I restriction enzyme reaction buffer, chromatin from 4×10^8^ cells was incubated with 300 units of RsaI for 3 hours at 37°C. Purified DNA from the digest was subjected to a secondary digestion by *Hae*III and then resolved on 1.5% agarose gel in 1×TAE buffer. Southern transfer of DNA to GeneScreen Plus Hybridization Transfer Membrane (Perkin Elmer) preparation was described previously [20].

### Preparation of radioactive probes for Southern blot analysis

These were undertaken as described in Teng *et al*, [20]. Details are available in Text S1.

### UV treatment of yeast cells, DNA isolation, and high-resolution mapping of CPD sites

These were undertaken as described by Reed *et al*, [31] and Teng *et al*, [32]. Details are available in Text S1.

## Supporting Information

Figure S1 Nucleosome positioning at the promoter of *MFA2*. Typical sequencing gels showing MNase-sensitive sites in the transcribed strand (TS, −412 to +83) and non-transcribed strand (NTS, −516 to −12) of the *Hae*III restriction fragment of *MFA2* in wild type and *tup1*Δ cells. The arrow indicates the transcription start site. The Mcm1 binding site and the TATA box are indicated. Nucleotide positions are allocated in relation to the *MFA2* start codon. Chromatin samples were treated with increasing amounts of MNase. For each set of chromatin samples (five lanes, left to right) the MNase concentrations used were 0, 1, 2, 5 and 10 U/ml. For naked DNA samples (three lanes, left to right) the MNase concentrations used were 2, 5 and 10 U/ml.
(TIF)

Figure S2 Repair of CPDs at the *MFA2* promoter. Gels depicting CPDs in the nontranscribed strand (NTS) of *Hae*III restriction fragment (−516 to −5) in the *MFA2* promoter in *rad16Δ*, *tup1*Δ*rad16Δ* and *tup1Δrad16Δgcn5*Δ cells, after 100 J/m^2^ UV irradiation. Lane U, DNA from unirradiated cells; lanes 0–4, DNA from irradiated cells after 0–4 hour of repair. Alongside the gels are symbols representing *MFA2* upstream activating sequences, Mcm1 binding site, and TATA box. Nucleotide positions are allocated in relation to the *MFA2* start codon.
(TIF)

Figure S3 Repair of CPDs at the *MFA2* promoter in wild-type (WT), rad16-K216A, rad16-C552AH554A, and rad16-K216AC552AH554A. Gels depicting CPDs in the nontranscribed strand (NTS) of *Hae*III restriction fragment (−446 to −21) in the *MFA2* promoter. Lane U, DNA from unirradiated cells; lanes 0–4, DNA from irradiated cells after 0–4 hour of repair. Alongside the gels are symbols representing *MFA2* upstream activating sequences, Mcm1 binding site, and TATA box. Nucleotide positions are allocated in relation to the *MFA2* start codon.
(TIF)

Figure S4 Repair of CPDs at the *MFA2* promoter of wild-type (WT), rad16-K216A, rad16-C552A,H554A, rad16-K216A,C552A,H554A, and rad16Δ strains. Time to remove 50% of the initial CPDs (T_50%_) at given sites. T_50%_ of a single CPD or a clustered group of CPDs with a similar repair rate was calculated (<4 hour) or extrapolated (>4 hour) as described previously (3). The T_50%_ of slowly repaired or unrepaired CPDs (T_50%_≥8 h) were represented at the 8 hour level on the graph. See also Figure S3.
(TIF)

Table S1 Plasmids and yeast strains used in this study.
(DOC)

Text S1 Supporting Discussion and Materials and Methods.
(DOC)
