# Supplementary figures and images for: How Chromatin Is Remodelled during DNA Repair of UV-Induced DNA Damage in Saccharomyces cerevisiae
Source: PLoS Genet. 2011 Jun 16;7(6):e1002124. doi: 10.1371/journal.pgen.1002124 (PMC3116912; doi:10.1371/journal.pgen.1002124)

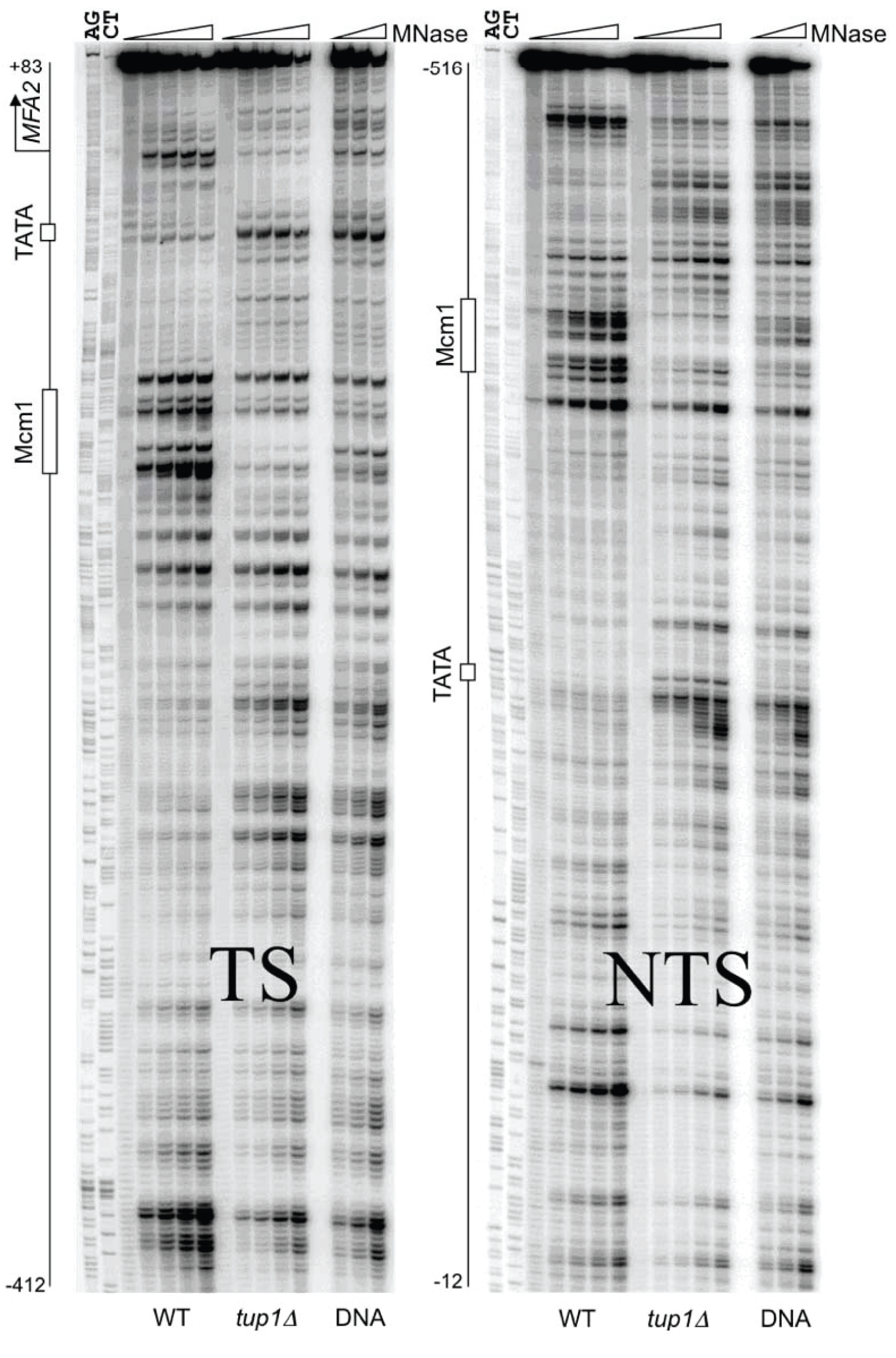

Supplement: Figure S1 — Nucleosome positioning at the promoter of MFA2. Typical sequencing gels showing MNase-sensitive sites in the transcribed strand (TS, −412 to +83) and non-transcribed strand (NTS, −516 to −12) of the HaeIII restriction fragment of MFA2 in wild type and tup1Δ cells. The arrow indicates the transcription start site. The Mcm1 binding site and the TATA box are indicated. Nucleotide positions are allocated in relation to the MFA2 start codon. Chromatin samples were treated with increasing amounts of MNase. For each set of chromatin samples (five lanes, left to right) the MNase concentrations used were 0, 1, 2, 5 and 10 U/ml. For naked DNA samples (three lanes, left to right) the MNase concentrations used were 2, 5 and 10 U/ml. (TIF) [file pgen.1002124.s001.tif]

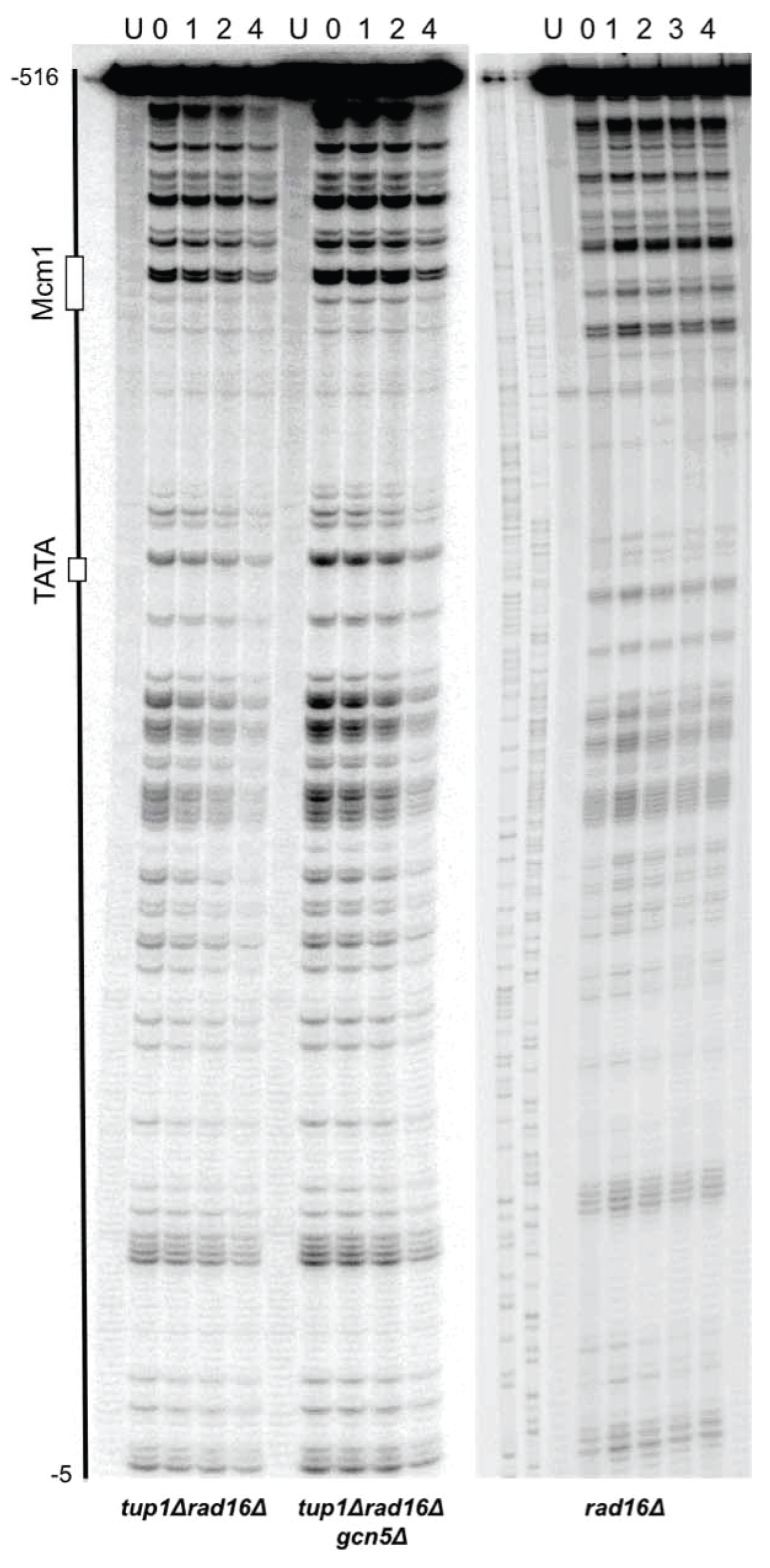

Supplement: Figure S2 — Repair of CPDs at the MFA2 promoter. Gels depicting CPDs in the nontranscribed strand (NTS) of HaeIII restriction fragment (−516 to −5) in the MFA2 promoter in rad16Δ, tup1Δrad16Δ and tup1Δrad16Δgcn5Δ cells, after 100 J/m2 UV irradiation. Lane U, DNA from unirradiated cells; lanes 0–4, DNA from irradiated cells after 0–4 hour of repair. Alongside the gels are symbols representing MFA2 upstream activating sequences, Mcm1 binding site, and TATA box. Nucleotide positions are allocated in relation to the MFA2 start codon. (TIF) [file pgen.1002124.s002.tif]

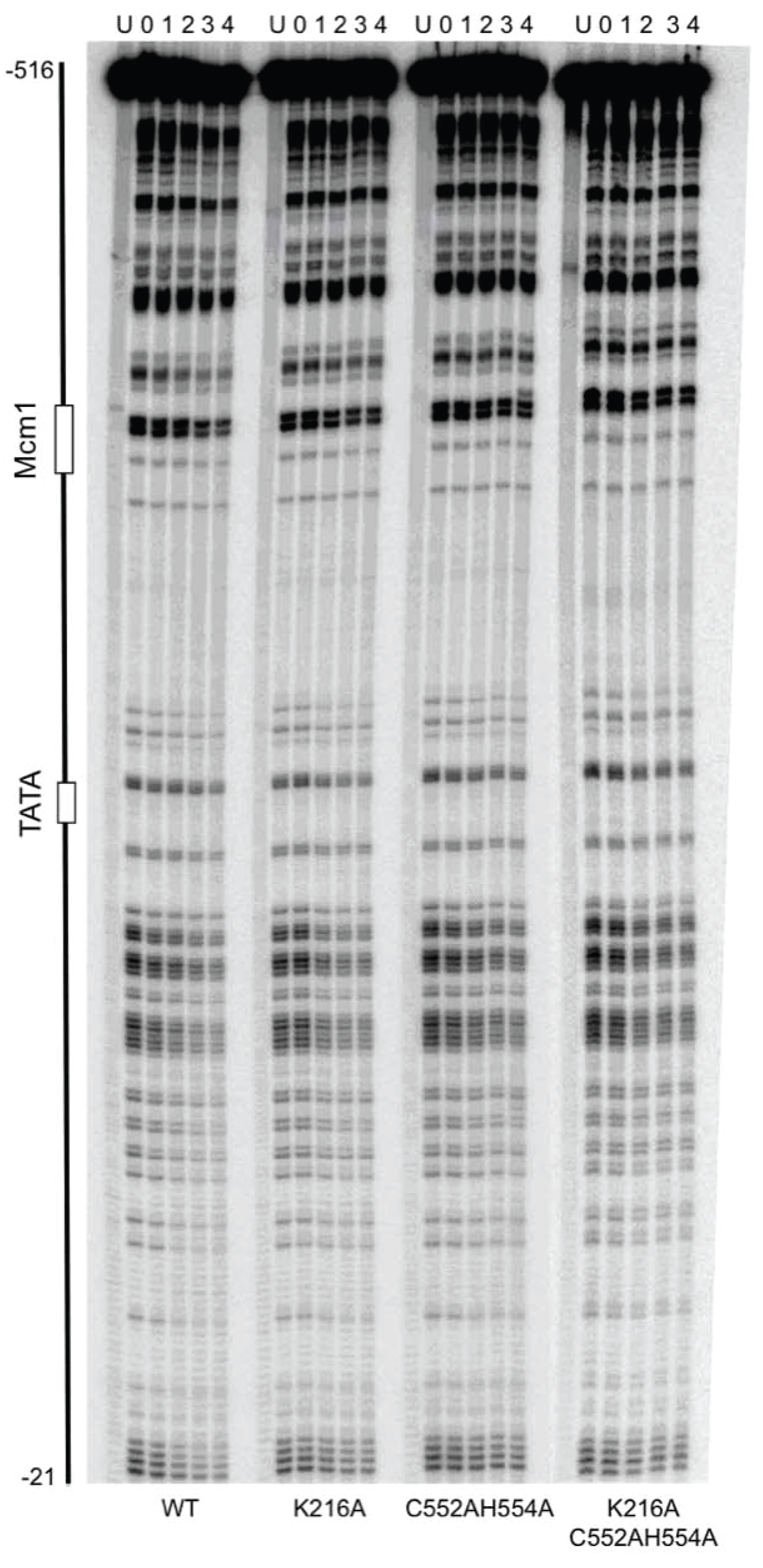

Supplement: Figure S3 — Repair of CPDs at the MFA2 promoter in wild-type (WT), rad16-K216A, rad16-C552AH554A, and rad16-K216AC552AH554A. Gels depicting CPDs in the nontranscribed strand (NTS) of HaeIII restriction fragment (−446 to −21) in the MFA2 promoter. Lane U, DNA from unirradiated cells; lanes 0–4, DNA from irradiated cells after 0–4 hour of repair. Alongside the gels are symbols representing MFA2 upstream activating sequences, Mcm1 binding site, and TATA box. Nucleotide positions are allocated in relation to the MFA2 start codon. (TIF) [file pgen.1002124.s003.tif]

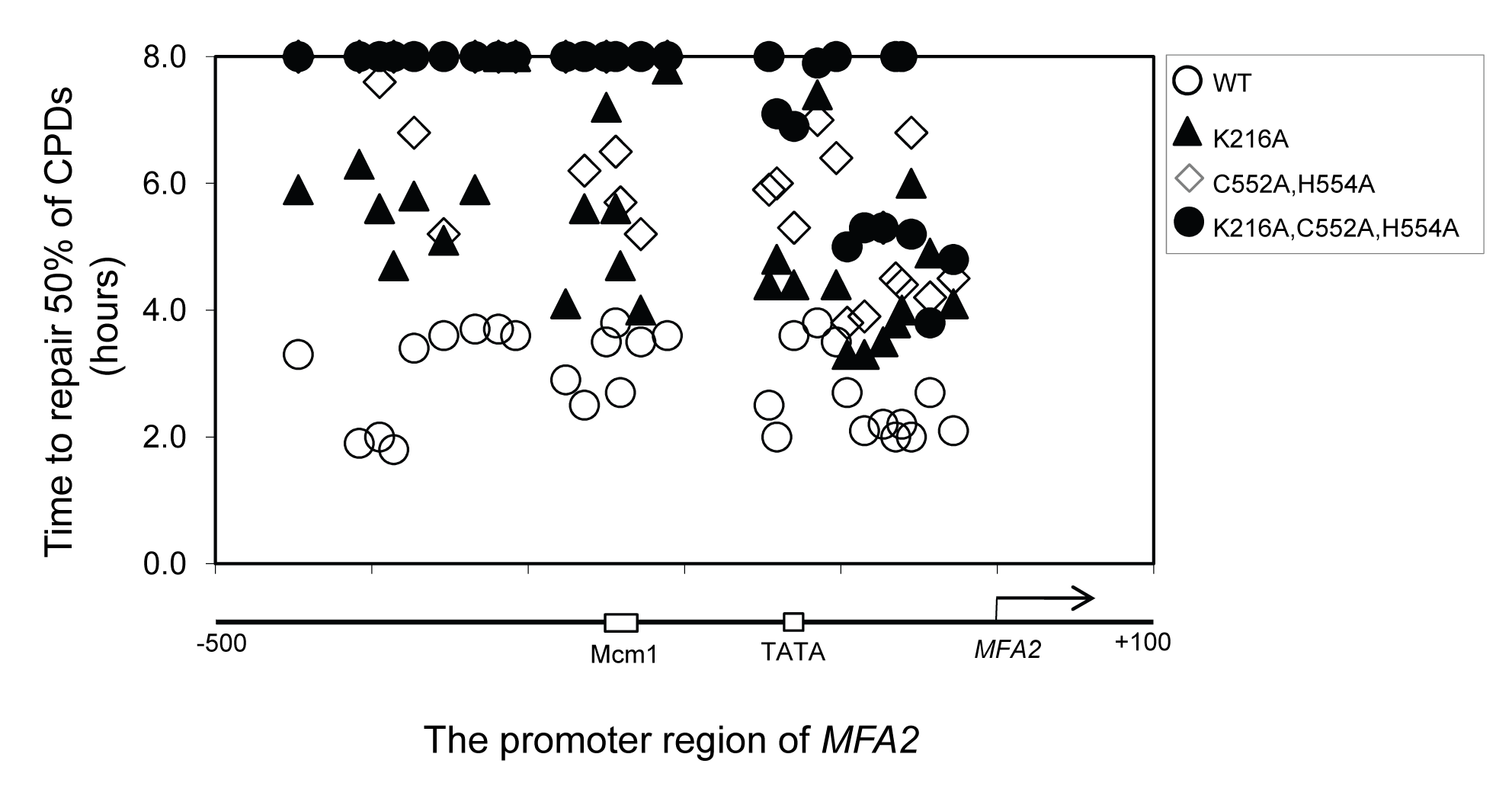

Supplement: Figure S4 — Repair of CPDs at the MFA2 promoter of wild-type (WT), rad16-K216A, rad16-C552A,H554A, rad16-K216A,C552A,H554A, and rad16Δ strains. Time to remove 50% of the initial CPDs (T50%) at given sites. T50% of a single CPD or a clustered group of CPDs with a similar repair rate was calculated (<4 hour) or extrapolated (>4 hour) as described previously (3). The T50% of slowly repaired or unrepaired CPDs (T50%≥8 h) were represented at the 8 hour level on the graph. See also Figure S3. (TIF) [file pgen.1002124.s004.tif]
