# Supplementary material for: How Chromatin Is Remodelled during DNA Repair of UV-Induced DNA Damage in Saccharomyces cerevisiae
Source: PLoS Genet. 2011 Jun 16;7(6):e1002124. doi: 10.1371/journal.pgen.1002124 (PMC3116912; doi:10.1371/journal.pgen.1002124)
Supplement: Table S1 — Plasmids and yeast strains used in this study. (DOC) [file pgen.1002124.s005.doc]

**Supporting Information Table S1.** Plasmids and yeast strains used in this study

| **Plasmids or yeast strains** | **Characteristics or Genotype** |
| --- | --- |
| pRAD16 | pRS315*-RAD16* |
| pK216A | pRS315*-RAD16K216A* |
| pC552AH554A | pRS315*-RAD16C552A,H554A* |
| pK216AC552AH554A | pRS315*-RAD16K216A,C552A,H554A* |
| PSY316 a | *MAT****a*** *ade2-101 ura3-52 leu2-3,112 Δhis3-200 lys2 trp1* |
| PSY316 α | *MAT*α *ade2-101 ura3-52 leu2-3,112 Δhis3-200 lys2 trp1* |
| PSY316*tup1Δ* | *MAT*α *ade2-101 ura3-52 leu2-3,112 Δhis3-200 lys2 trp1tup1Δ::HIS3* |
| PSY316*gcn5Δ* | *MAT*α *ade2-101 ura3-52 leu2-3,112 Δhis3-200 lys2 trp1* |
| PSY316*rad16Δ* | *MAT*α *ade2-101 ura3-52 leu2-3,112 Δhis3-200 lys2 trp1* *rad16Δ::URA3* |
| PSY316*rad7Δ* | *MAT*α *ade2-101 ura3-52 leu2-3,112 Δhis3-200 lys2 trp1rad7Δ::TRP1* |
| PSY316*tup1Δrad16Δ* | *MAT*α *ade2-101 ura3-52 leu2-3,112 Δhis3-200 lys2 trp1tup1Δ::HIS3 rad16Δ::URA3* |
| PSY316*tup1Δrad7Δ* | *MAT*α *ade2-101 ura3-52 leu2-3,112 Δhis3-200 lys2 trp1tup1Δ::HIS3 rad7Δ::TRP1* |
| PSY316*tup1Δ gcn5Δ* | *MAT*α *ade2-101 ura3-52 leu2-3,112 Δhis3-200 lys2 trp1tup1Δ::HIS3* |
| PSY316*tup1Δrad16Δgcn5Δ* | *MAT*α *ade2-101 ura3-52 leu2-3,112 Δhis3-200 lys2 trp1 tup1Δ::HIS3 rad16Δ::URA3* |
| BY4742 (WT) | *MAT*α *his3Δ1 leu2Δ0 lys2Δ0 ura3Δ0* |
| BY4742*rad16Δ* | *MAT*α *his3Δ1 leu2Δ0 lys2Δ0 ura3Δ0 rad16Δ::kanMX4* |
| BY4742*rad7Δ* | *MAT*α *his3Δ1 leu2Δ0 lys2Δ0 ura3Δ0* *rad7Δ::kanMX4* |
| BY4742*GCN5/myc* | *MATα his3Δ1 leu2Δ0 lys2Δ0 ura3Δ0 GCN5::myc9-URA3* |
| BY4742*rad16ΔGCN5/myc* | *MATα his3Δ1 leu2Δ0 lys2Δ0 ura3Δ0 rad16Δ:: kanMX4 GCN5::myc9-URA3* |
| BY4742*rad7ΔGCN5/myc* | *MATα his3Δ1 leu2Δ0 lys2Δ0 ura3Δ0 rad7Δ::kanMX4 GCN5::myc9-URA3* |
| BY4742*GCN5/myc* p*RAD16* (WT) | *MATα his3Δ1 leu2Δ0 lys2Δ0 ura3Δ0 rad16Δ:: kanMX4 GCN5::myc9-URA3* pRAD16 |
| BY4742*GCN5/myc* p*K216A*  (*rad16-K216A*) | *MATα his3Δ1 leu2Δ0 lys2Δ0 ura3Δ0 rad16Δ:: kanMX4 GCN5::myc9-URA3* pK216A |
| BY4742*GCN5/myc* p*C552A,H554A* (*rad16-C552A,H554A*) | *MATα his3Δ1 leu2Δ0 lys2Δ0 ura3Δ0 rad16Δ:: kanMX4 GCN5::myc9-URA3* pC552AH554A |
| BY4742*GCN5/myc* p*K216A,C552A,H554A* (*rad16-K216A,C552A,H554A*) | *MATα his3Δ1 leu2Δ0 lys2Δ0 ura3Δ0 rad16Δ :: kanMX4 GCN5::myc9-URA3* pK216AC552H554A |
