# Supplementary material for: How Chromatin Is Remodelled during DNA Repair of UV-Induced DNA Damage in Saccharomyces cerevisiae
Source: PLoS Genet. 2011 Jun 16;7(6):e1002124. doi: 10.1371/journal.pgen.1002124 (PMC3116912; doi:10.1371/journal.pgen.1002124)
Supplement: Text S1 — Supporting Discussion and Materials and Methods. (DOC) [file pgen.1002124.s006.doc]

### SUPPORTING INFORMATION

**Supporting Discussion**

In wild type cells in the absence of UV damage (Figure 7 upper panel), only basal levels of Gcn5 occupancy at the promoter region of *MFA2* are observed, consequently the histone H3 acetylation status is maintained at a low level, and chromatin remains in a closed configuration preventing gene transcription from *MFA2*. Following UV irradiation (Figure 7 lower panel), Rad7 and Rad16 dependent increased occupancy of the histone acetyl transferase Gcn5 (Figure 7 (3)) results in elevated levels of histone H3 acetylation (Figure 7 (4)). This promotes an open chromatin structure at *MFA2* (Figure 7 (5)). Our results show that this is achieved via the dual action of the DNA translocase (Figure 7 (1)) and E3 ligase (Figure 7 (2)) activities associated with the ATPase and RING domains of the Rad16 component of the GG-NER complex. We recently showed that the GG-NER complex exhibits DNA translocase activity associated with the ATPase domain of Rad16. This activity generates superhelical torsion in DNA necessary for excision of DNA damage during GG-NER *in vitro*. In the same study we also reported that the GG-NER complex is not able to slide nucleosomes in an *in vitro* assay, unlike many SWI/SNF chromatin remodelling complexes. We suggest that the DNA translocase activity of the GG-NER complex specifically modifies chromatin structure to facilitate access of the Gcn5 histone acetyl transferase, which subsequently promotes an open chromatin structure necessary for efficient GG-NER via increased histone H3 acetylation in the region. We reported previously that during GG-NER at *MFA2*, the gene remains repressed. We suggest that failure of the GG-NER complex to slide nucleosomes at the promoter of *MFA2* in response to UV prevents access to critical transcription initiation sites such as the TATA box, which precludes the sequence specific binding of key transcription initiation factors. In this way, the GG-NER complex can promote specific changes in chromatin structure required for efficient GG-NER (Figure 7 (7)), while at the same time preventing unregulated gene transcription (Figure 7 (6)). Our results also reveal the importance of the RING domain of Rad16 in promoting chromatin remodelling necessary for efficient GG-NER. This suggests that the E3 ubiquitin ligase activity of the GG-NER complex also plays a role in chromatin remodelling necessary for repair.

**Supporting materials and methods**

***Plasmids and yeast strains***

The plasmids used in this study are described in Supporting Information Table S1. *CEN ARS* plasmids encoding *RAD16* was constructed by PCR and standard subcloning procedures. Creation of the point mutations within the Rad16 ATPase domain (K216A), RING-finger domain (C552A,H554A) and double mutation of ATPase and RING-finger domains (K216A,C552A,H554A), resulting in the amino-acid substitutions, lysine 216 to alanine, cysteine 552 to alanine and histidine 554 to alanine, were achieved by site-directed mutagenesis of the wild-type *RAD16* gene cloned in pRS315 (p*RAD16*) using Stratagene QuickChange Lightning site-directed mutagenesis system as recommended by the manufacturer. Mutations were confirmed by sequencing. The plasmids and yeast strains used in this study are listed in Supporting Information Table S1. The cells were grown in YPD or Synthetic Complete Leucine dropout medium (SC-Leu).

***Epitope tagging of yeast strains***

Gcn5 was tagged at the C-terminus by inserting 18 copies of the Myc epitope coding sequence into the normal chromosomal loci of *GCN5*. The plasmid p3747, a kind gift from Dr. Richard A Young, was used as a template to generate PCR products containing the Myc epitope coding sequence and URA3 selectable marker flanked by homologous regions designed to recombine at the 3′ end of the *GCN5*. The PCR products were transformed into the BY4742, and its derivatives *rad16Δ* and *rad7Δ* mutant strains. Clones were selected for growth on URA^-^ selective plates. Insertion of the epitope coding sequence was confirmed by PCR and sequencing, and the expression of the epitope-tagged protein was confirmed by Western blotting using an anti-Myc antibody.

***UV treatment of yeast cells and DNA isolation***

These were undertaken as described by Reed *et al* (1). In brief, exponential phase yeast cells were resuspended at 2×10^7^ cells/ml in pre-chilled phosphate buffered saline (PBS) and irradiated with 254 nm UV light at a dose of 100 J/m^2^. Irradiated cells were collected by centrifugation, resuspended in fresh YPD, and incubated with shaking for various repair times in the dark at 30°C prior to DNA isolation. For DNA isolation, yeast cells were first treated with zymolyase to produce spheroplasts, which were then efficiently lysed with sodium dodecyl sulphate solution. Following RNase A and proteinase K treatment, the cell lysate was extracted twice with phenol/chloroform and once with chloroform, and the DNA was precipitated with ethanol.

***High resolution mapping of the CPD sites***

This was carried out as previously described (2). In brief, 30 µg of DNA was digested with *Hae*III at 37^o^C for 2 hours in a total reaction volume of 200 μl generating a 599 bp fragment covering *MFA2* promoter and part of the transcribed region. The enzyme was removed by phenol/chloroform extraction and the DNA was then precipitated using 20 µl of 3M NaAc and 220 µl of isopropanol. After centrifugation, the DNA pellets were resuspended in 100 µl of TE buffer. The DNA samples were cleaved specifically at CPD sites using T4 endonuclease V according to manufacturer’s recommendations. T4 endo V was removed by phenol/chloroform extraction followed by chloroform extraction. To the samples, 1 pmol of biotinylated probe 1 was added (5’biotin-GATAGCTTTTTTCCCTCATCTATTTTCTCGGAAAACTTGGTG3’) to detect damage in the transcribed strand (TS). The samples were denatured at 95°C for 5 min and then incubated at 55°C for 15 min to allow the probe to anneal to the *MFA2* fragments. To each sample 100 µg of washed Dynabeads M-280 Streptavidin (Invitrogen) was added and mixed well. The mixtures were kept at room temperature for 15 min to allow the streptavidin-coated Dynabeads to bind to the biotin molecule at the 5’-end of the probe. The tubes were placed in a Dynal magnetic particle concentrator (MPC) and the beads with the attached *MFA2* fragments were attracted to the wall of the tube. The MPC was used at subsequent steps to attract the fragments to the wall of the tube following the various washing and labelling steps. The supernatant containing the NTS fragments and other genomic DNA fragments was removed to a fresh tube for purification of the *MFA2* non-transcribed strand (NTS) following the same procedure as for the TS except using probe 2 (5’biotin- GATAGCTTTTTTCCCTTGATTATATAGATTGTCTTTCTTTTCAG AGGAT3’). The beads bound with *MFA2* fragments were washed with 50 µl of washing buffer (5 mM Tris–HCl, pH 7.5, 0.5 mM EDTA, 1.0 M NaCl) at 55°C for 5 min and the buffer removed. The second and third washes were performed at room temperature with 50 µl of water. The *MFA2* fragments were end-labelled with six [α-^32^P] dATP residues by T7 DNA polymerase (Amersham) using the six-T overhang in the probe as a template. The labelling reaction was complete after 10 min at 37°C. The labelling medium was removed while the beads with the bound *MFA2* fragments were kept on the wall of the Eppendorf tube by the MPC. Following two washes with 50 µl of TE, the labelled *MFA2* fragments were eluted from the Dynabeads at room temperature by the addition of 3 µl of formamide loading buffer (95% formamide, 20 mM EDTA, 0.05% bromophenol blue). The eluted fragments were resolved by electrophoresis on a 6% denaturing polyacrylamide gel. Electrophoresis was carried out in a field of 40 V/cm in TBE (89 mM Tris base, 89 mM boric acid, 2.5 mM Na_2_EDTA•2H_2_O), and the image was revealed by phosphorimaging.

***The determination of DNA damage and repair***

We quantified the signal for the full length DNA band and all detectable damaged bands. The total signal in a lane is obtained by summing the measurements over the entire lane, which is then used to normalize the amount of DNA used for each lane. It is important to note that many repair trends cannot be easily distinguished by visual examination of these gels. Up to 50% variation can occur in loadings per lane. Thus repair is only quantifiable by analysis of total radioactivity versus that in given bands of a lane as estimated from the phosphorimage.

***Preparation of radioactive probes for southern blot***

The primers used to amplify the *Hae*III digested *MFA2* DNA fragments are 5’-biotin-GATAGCTTTTTTCCCTTGATTATATAGATTGTCTTTCTTTTCAGAGGAT-3’ (Primer A) and 5′-GATAGCACACCATCTACTACATAATTAATTGATAGT-3′ (Primer B). Standard PCR was carried out using Primers A and B to amplify the sequences of interest. 20 μl of washed Dynabeads M-280 Streptavidin (Invitrogen) was added to 5 µl of PCR product to bind to the biotin at the 5' end of the extended Primer A. The Dynabeads with the associated PCR product were collected using a MPC and the supernatant was discarded. The Dynabeads were resuspended in 20 μl of 0.1 N NaOH to denature the dsDNA. The biotinylated strand associated with the beads was collected using a MPC and washed twice with 50 µl of H_2_O, then resuspended in 10 µl of H_2_O with addition of 2 pmol of Primer B. The tube was incubated at 65°C for 2 min, then allowed to slowly cool to room temperature in a water bath. The beads and bound fragments were washed twice with 50 μl of water and resuspended in 15 μl of water, 6 µl of 5× Sequenase reaction buffer (Amersham), 4 µl of dNTP buffer (0.1 mM dTTP, dGTP and dCTP), 2 µl of [^32^P] dATP (6000 Ci/mmol, Amersham), 2 µl of DTT (0.1M), 1 µl of Sequenase (Amersham). Primer B was extended by using the biotinylated strand as a template, at 37°C for 10 min. The beads with the associated dsDNA fragment were washed twice with 50 µl of water, and then resuspended in 100 µl of TE buffer. After heating at 95°C for 5min, the supernatant containing the synthesised radioactive strand (probe) was separated from the beads and transferred into hybridization tubes.

**Supporting references**

1. Reed SH, Boiteux S, & Waters R (1996) UV-induced endonuclease III-sensitive sites at the mating type loci in Saccharomyces cerevisiae are repaired by nucleotide excision repair: RAD7 and RAD16 are not required for their removal from HML alpha. (Translated from eng) *Mol Gen Genet* 250(4):505-514 (in eng).

2. Teng Y, Li S, Waters R, & Reed SH (1997) Excision repair at the level of the nucleotide in the Saccharomyces cerevisiae MFA2 gene: mapping of where enhanced repair in the transcribed strand begins or ends and identification of only a partial rad16 requisite for repairing upstream control sequences. (Translated from eng) *J Mol Biol* 267(2):324-337 (in eng).

3. Teng Y, Yu Y, & Waters R (2002) The Saccharomyces cerevisiae histone acetyltransferase Gcn5 has a role in the photoreactivation and nucleotide excision repair of UV-induced cyclobutane pyrimidine dimers in the MFA2 gene. (Translated from eng) *J Mol Biol* 316(3):489-499 (in eng).
